# Early disruptions to syringe services programs during the Russian invasion of Ukraine

**DOI:** 10.3389/fpubh.2023.1229057

**Published:** 2023-11-21

**Authors:** Benjamin M. Nikitin, Daniel J. Bromberg, Iryna Pykalo, Roman Ivasiy, Zahedul Islam, Frederick L. Altice

**Affiliations:** ^1^Yale School of Medicine, New Haven, CT, United States; ^2^Ukrainian Institute on Public Health Policy, Kyiv, Ukraine; ^3^International Alliance for Public Health Ukraine, Kyiv, Ukraine

**Keywords:** Ukraine, war, harm reduction, HIV, syringe services programs, implementation science

## Abstract

**Introduction:**

The widespread HIV epidemic in Ukraine is concentrated among people who inject drugs (PWID), making access to sterile injection paraphernalia (SIP) like sterile needles and syringes a critical method of HIV/AIDS prevention; however, the Russian invasion has threatened to disrupt the operations of syringe services programs (SSPs), creating a risk of HIV outbreaks among PWID.

**Methods:**

We conducted 10 semi-structured interviews with outreach workers from SSPs. Interviews were purposively sampled to cover three prototypic regions of Ukraine: temporarily Russian-controlled, frontline, and destination. Qualitative results from interviews were then compared against a standardized, nationwide harm reduction database.

**Results:**

We found that the Russian invasion triggered both supply and demand challenges for SSPs. Demand increased for all regions due to client transitions from pharmacies that closed to SSPs, increases in illicit drug use, greater client openness to NGO support, and displacement of clients to destination regions. Supply decreased for all areas (except for remote destination regions) due to battle-related barriers like curfews, roadblocks, and Internet disruptions; diminished deliveries of SIP and funding; and staff displacement. Time series plots of the number of unique clients accessing harm reduction services showed that an initial decrease in service provision occurred at the start of the war but that most regions recovered within several months except for Russian-controlled regions, which continued to provide services to fewer clients relative to previous years.

**Conclusion:**

To ensure continued scale-up of SIP and other HIV prevention services, the SyrEx database should be leveraged to serve as a streamlined harm reduction locator that can inform workers and clients of open site locations and other pertinent information.

## Introduction

Ukraine has the second-largest HIV epidemic in Europe, with ~360,000 people with HIV (1) and most cases being concentrated among people who inject drugs (PWID); over 1 in 5 PWID currently have HIV (2). Moreover, prevalence of PWID is substantially higher than observed throughout Europe, with 1.4% Ukrainians being PWID (3).

Syringe services programs (SSPs) substantially reduce risk of HIV transmission in PWID through distribution of sterile injection paraphernalia (SIP) along with disinfectant, provision of other tools for safe injection and linkage to opioid agonist therapies (4). Unlike pharmacies, which also dispense SIP, SSPs are generally free of charge and offer an array of other services like HIV screenings and linkage to addiction treatment (5). SSPs were introduced in Ukraine primarily through funding from the Global Fund to Fight AIDS, Tuberculosis, and Malaria (GFATM), which was directed to the NGO Alliance for Public Health and their distribution of partners throughout Ukraine, including the non-occupied parts of Donetsk and Luhansk after 2013. SSPs remain a crucial component of Ukraine’s national HIV response (6). Sterile syringes may also be purchased at pharmacies (6).

Harm reduction organizations in Ukraine manage SSPs at both brick-and-mortar and through mobile outreach delivery (7). These efforts are funded by the Ukrainian government and GFATM and its recipient non-governmental organizations in Ukraine (8). Prior to the 2022 invasion, over 135 harm reduction organizations received funding to provide SSP services (9). SSPs are primarily operated by outreach workers (sometimes referred to as SSP workers); outreach workers not only provide direct SSP services but also help clients navigate the prevention and treatment healthcare system (10). Scale-up of SSPs is a highly effective method for preventing the spread of HIV among PWID and between their sexual partners (4). Prior to the Russian invasion of Ukraine in 2022, over one-third of PWID without HIV accessed SSPs annually (11). The 2022 Russian invasion of Ukraine, however, markedly disrupted HIV prevention and treatment services in the country, including opioid agonist maintenance therapies (OAMT) and antiretroviral therapy (12). Site closures early in the war related to conflict and internal displacement of over 6.5 million Ukrainians have led to client and provider displacement, exacerbating disruptions to these services (13). UNAIDS estimates that nearly one-third of all people with HIV (PWH) receiving treatment experienced interruptions in their treatment since the start of the Russian invasion (14). Considering these disruptions, the conflict has likely caused similar challenges to the scale-up of SSPs in Ukraine. Sustaining scale-up is particularly important during this period, as PWID are especially sensitive to stressors like war, which in turn can result in exacerbated HIV transmission. This risk is further increased due to displacement of PWID within Ukraine and to destinations across Europe (14).

Here, we examine the extent to which SSPs in Ukraine evolved and altered their implementation strategies during the early phases of the Russian invasion. We used a previously described conflict framework that has been used to understand implementation of OAMT to analyze how different regions of the country responded to harm reduction during the early invasion (12, 15). Specifically, discontinuing OAMT has immediate adverse consequences on health, namely abstinence syndrome, overdose, and death (16), as observed in the annexation of Crimea (17), while changes in SSP delivery are likely to have immediate consequences for transmission of HIV and HCV. Moreover, disruptions in OAMT can result in increased needs for SIP. Despite these risks, SSPs have received minimal mentions in prior research on harm reduction programs in Ukraine (18, 19), especially during the war. Based on these observed research gaps, we designed this study to evaluate how SSPs in Ukraine have been affected during the war, especially based on their proximity to conflict. Based on these findings, we then considered how scale-up of SSP services can be sustained despite war-time disruptions.

## Methods

In this mixed methods study, we employed qualitative analysis of semi-structured interviews and quantitative analysis of harm reduction data to evaluate the impact of the Russian invasion on SSPs.

### Study design

**Figure 1:**
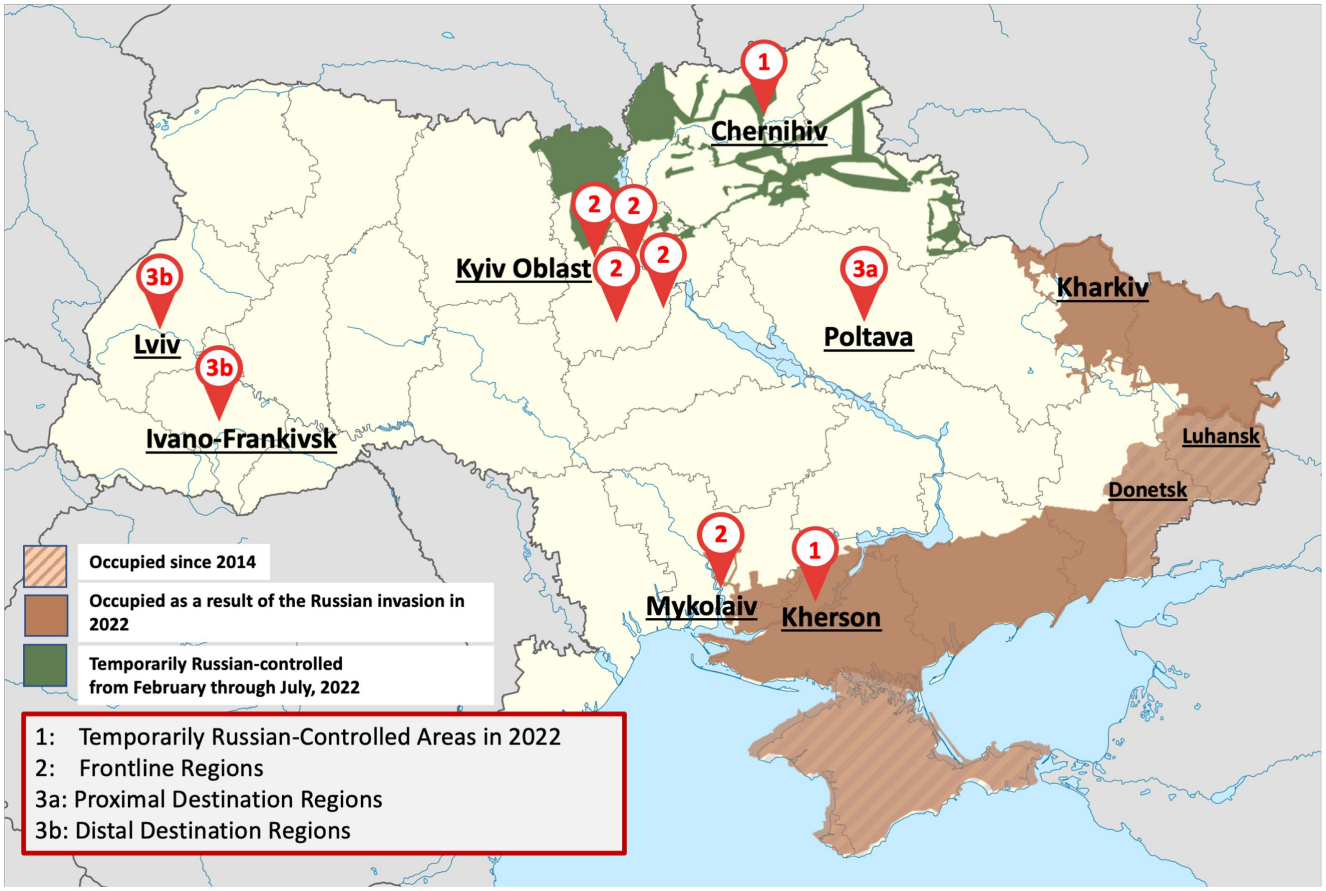
Map of Ukraine depicting sites where interviews were performed and the prototypical classification of each of these sites. Russian-occupied areas are also identified.

Due to the unique nature of the conflict, we analyzed data using a risk framework for Ukraine based on the proximity of areas to conflict and their relationship to OAMT delivery (15). This framework was modified, as the conflict regions have evolved throughout the war. We categorized the regional responses based on the perceived experience of SSP providers within each region as well as data on internal displacement. Based on these metrics, we categorized regions using three distinct prototype regions: temporarily *Russian-controlled*, or areas where Russian forces had taken a form of control (e.g., roadblocks or full government control) over part of the region; *frontline*, or areas that were exposed to battle-related conflict but were not Russian-controlled; and *destination*, or areas that displaced persons had settled in for over 3 months after coming from Russian-controlled and/or frontline regions. Though most destination regions were in western Ukraine (distal), others were more central, like Poltava (proximal; Figure 1).

We further categorized our analysis based on supply and demand dynamics observed by SSP providers. We derived definitions for supply and demand in the context of SSP inductively from qualitative data, and these categories served only as comparative metrics for pre-vs. post-war dynamics rather than quantitative metrics. We defined demand as client-side changes in requests for access to SIP and other SSP services. We defined supply as changes in the capacity of SSPs to provide SIP and related services to clientele.

### Sampling and study participants

From May through July 2022, we performed semi-structured interviews (*N* = 10; 45 min each) with outreach workers from harm reduction organizations that manage SSPs within one of these three distinct regions. Interviews were completed in Russian by two coauthors, BMN and DJB, using video-conferencing software. Interview participants were purposively sampled based on the outlined theoretical framework to include similar numbers of participants from three conceptual areas based on proximity to the conflict: temporarily Russian-controlled, frontline, and destination regions. A semi-structured interview guide was used to direct questions, which probed participants on changes to supply and demand dynamics for SSPs following the Russian invasion of Ukraine (see Supplementary material); the interview guide evolved slightly over time. Interviews were transcribed verbatim, translated, and back-translated for content in real-time (20) and interviews were stopped when thematic saturation was achieved.

### Data analysis

After de-identifying translated transcripts, BMN and DJB coded the transcripts in NVivo (Release 1.7.1) and where coding differed, a third coauthor arbitrated the disagreement. Quantitative harm reduction data were provided by the Alliance for Public Health from the SyrEx Database, which is used by harm reduction organizations in Ukraine to record the quantity of clients they serve and the services they provide. Data from SyrEx did not include the quantity of SIP distributed to clients. Therefore, we applied data detailing the number of unique clients among PWID who received at least one preventive service over time as a proxy for the quantity of clients that were specifically provided SIP. We generated time series of changes to this metric over time, from January 2019 through September 2022. Time series were analyzed for the nationwide aggregate of clients as well as for each Ukrainian oblast (province) in the study to visualize whether scale-up of SSP and harm reduction services was sustained over time, particularly after 24 February 2022.

### Ethics

This study was approved by institutional reviews boards (IRBs) at Yale University and the Ukrainian Institute on Public Health Policy. Due to the low-risk nature of the study and the ongoing conflict in Ukraine, both IRBs endorsed collection of verbal consent from participants. All identifying information described by interview participants was redacted prior to transcription. All participants were financially compensated for interviews.

## Results

We interviewed 10 SSP workers from harm reduction organizations in Ukraine, located in temporarily Russian-controlled (*N* = 2), frontline (*N* = 5), and destination (*N* = 3) regions, as per our framework. From these interviews, we observed that SIP distribution during the war was disrupted due to changes in both supply and demand of these services, with considerable variation across regions.

### Demand-side changes: “They are waiting for us like manna from heaven”

For all three prototype regions, participants reported that demand for SSPs had increased after the 2022 Russian invasion.

#### Displacement of clients within Ukraine

Due to violent conflict in temporarily Russian-controlled regions, participants located in frontline and destination regions observed an influx of displaced SSP clients from the Russian-controlled regions. When conflict was pronounced throughout the country at the start of the war, destination regions observed a rapid influx of displaced persons from frontline regions as well. In Ivano-Frankivsk, an SSP worker reported that his organization had begun supporting over 40 new clients at the start of the war; however, with violent conflict later becoming concentrated in Russian-controlled regions, many displaced persons left destination regions to return to their homes in frontline regions, including Kyiv. Within many frontline regions themselves, internal client displacement from Russian-controlled regions was also observed by participants. An SSP worker in Kaharlyk, Kyiv Oblast, indicated that the quantity of displaced clients from other regions led to a 30% increase in their overall client base. This increase in new clients due to internal displacement increased the workload of most organizations in these regions, though SSP workers generally reported that the quality of their work had not been impacted despite the increased workload.

*“Everything [in SSPs] works, just more work has been added [due to internally displaced clients]”* (Lviv, destination).

Clients were displaced from Russian-controlled regions at the highest rate, though many remained, and newly displaced clients emerged in these regions as well. While controlled by Russia, the region was locked down, and individuals were not permitted to leave. Even prior to this closure, however, many clients chose to remain due to financial barriers and/or family issues.

*“To go somewhere else, you need to have money, you need to have some kind of profession to somehow live there, to have somewhere to go”* (Kherson, Russian-controlled).

In other cases, the release of prisoners due to an influx of pardons at the start of the Russian invasion caused an increase in the number of PWID in the region seeking access to HIV prevention services, including SSPs.

*“There were amnesties for people who were serving sentences in camps and prisons. And so, those who did not have time to leave settled [in Kherson]. It's hard for them. And so, they come to us”* (Kherson, Russian-controlled).

#### PWID transition from pharmacies to SSPs

Prior to the invasion, many PWID preferred purchasing SIP in pharmacies rather than obtaining it from SSPs due to distrust of these programs. In the initial weeks of the invasion, however, many pharmacies across the country closed, leading PWID who had previously relied on pharmacies to pivot to SSPs, most of which continued operating.

*“In the past, if customers could get a job, they could get clean syringes or alcohol wipes and stuff like that [from pharmacies], but now our customers are out of work. Why? Because the small businesses, including pharmacies, have closed”* (Mykolaiv, frontline).

Even as pharmacies reopened, PWID continued to rely on SSPs due to financial constraints. The costs of syringes in pharmacies increased substantially. In Russian-controlled and frontline regions, small business closures were common and often permanent, leading many PWID to lose their sole source of income. Even after pharmacies reopened following reductions in violence, many PWID lacked the funds to purchase SIP from pharmacies, resulting in increased reliance on SSPs. In response to the war, some pharmacies hiked up syringe prices, resulting in an additional financial barrier to access.

*“It was noticed that people who used to buy syringes themselves in pharmacies started saving money and coming to us to buy syringes. Because the money they were saving, they were using it to … buy drugs”* (Poltava, proximal destination).

*“As far as I know, syringes have … doubled and tripled in price”* (Lviv, distal destination).

#### Increases in illicit drug use

Loss of employment also resulted in increased stress and desperation among SSP clients, many of whom had substance use disorders that made them more vulnerable to stress (21). This stress, even for clients who kept their jobs, was compounded by persistent violence, leading many to increase injection drug use. Across Ukraine, interviewed participants hypothesized that these stressors had markedly increased with emerging trends in the drug use environment, including expanded use of synthetic cathinones (bath salts) and diphenhydramine (Dimedrol) as opioids became less available. Synthetic cathinones are injectable stimulants that are less expensive to purchase in the black market than other stimulants, while diphenhydramine is an over-the-counter antihistamine that is injected to enhance the effects of other substances, like methadone. The heightened use of these injectable substances during the war, compounded by minimal access to pharmacies, caused an increase in demand for SSPs.

*“[After the war] a lot of people have started to use bath salts. They are probably cheaper … We need to give out more [SIP than before]”* (Lviv, distal destination).

*“Dimedrol is now one of the most popular products in pharmacies… It seems like two-thirds of my clients now use Dimedrol, they say they need [larger] needles and syringes”* (Mykolaiv, frontline).

*“I honestly know that a lot of new people use Dimedrol … They’re now supplementing with it because they say that our methadone is weak”* (Ivano-Frankivsk, distal destination).

Aside from wartime stresses related to violence and financial issues, some clients increased their use of substances like synthetic cathinones and diphenhydramine during the war due to disruptions in OAMT services, such as when clinics closed and/or when OAMT clinics chose to reduce dosages of OAMT out of concern for insufficient medication supply.

Limited access to methadone in clinics as well as to buprenorphine (which is generally dispensed in pharmacies) likely led PWID to resort to purchase of illegal opioids like “street methadone,” another unknown opioid. Unlike methadone prescribed in clinics, which must be consumed orally, “street methadone” is usually injected by PWID, increasing the risk of transmission of HIV and HCV. This change in access has likely increased the demand for SIP among SSP clients.

*“[OAMT clinics] started to give them [medication] less often. They used it all very quickly and then they sat without anything. Or they had to buy street drugs, and there was no money for that. Because if they could earn extra money somewhere else in peacetime, during the war it was very hard for them”* (Bila Tserkva, Kyiv Oblast; frontline).

*“It was impossible to get to Kyiv from Boyarka [to receive OAMT]… there were problems for a month … [The clients] switched to ‘street methadone’ [because of these difficulties], and ‘street methadone’ is of very poor quality.”* (Boyarka, Kyiv Oblast; frontline).

#### Evolving client sentiment

The severity of the Russian invasion, compounded by the desperation experienced by SSP clients, appeared to make clients more receptive to accept help from harm reduction organizations, despite client trust remaining limited.

*“People are open now to any help. And they are more open to such services [like SSP], to* any *services that are provided”* (Chernihiv, Russian-controlled).

This change in client attitude further increased demand.

### Supply-side changes

Though SSP supply remained relatively constant in distal destination regions since the Russian invasion, it markedly decreased in both Russian-controlled frontline regions and proximal destination regions. In these areas, despite heightened demand for SIP, SSPs encountered major barriers to delivering those services to clients.

#### Staff displacement

As with clients, many SSP workers from harm reduction organizations were displaced due to conflict, with many moving to destination regions in Ukraine and/or neighboring countries in Europe. Though displaced staff generally continued to work remotely, SSPs lost in-person staffing capacity, which was critical for maintaining contact with clients and ensuring that clients had continuous and sufficient access to SIP. One participant reported that each SSP worker managed a specific set of clients within their organization; the loss of in-person staff in their organization led them to lose contact with several clients.

*“Every SSP worker has a client base. So, these are the people he’s been in contact with for five to seven years. Because if we take our organization, we have people working for us for fourteen, fifteen, sixteen years … We suffer a lot when someone leaves our organization. These are people who have been working [with their clients] for a long time, who have [experience with them], and they know each other”* (Mykolaiv, frontline).

Most organizations managed to hire new staff. For newly hired SSP workers, however, harm reduction organizations needed to ensure that they were adequately trained and had built sufficient trust with clients, a process that could take up to several months. The rapid turnover of staff led to substantial strains on harm reduction organizations, which reduced the supply capacity of SSPs by extension.

*“Four people [left]. One joined us. Well, of course, the workload on the rest of the SSP workers increased. Work has increased for the entire staff, because if earlier four more people did it, now these four are gone, and the work remains”* (Kherson, Russian-controlled).

#### Curfews and roadblocks

In some Russian-controlled and frontline regions, the imposition of roadblocks, curfews, and other battle-related disruptions inhibited organizations from routine distribution of SIP. The enforcement of strict curfews forced organizations to decrease their working hours, particularly during the most violent periods of the war.

*“Where I work, there was shelling nearby. There were troops landing nearby. And that’s why we were handing out supplies very fast, two or three hours of work and that’s it”* (Obukhiv, Kyiv Oblast; frontline).

For organizations operating in regions with restrictive roadblocks, they encountered major logistical barriers in their capacity to distribute SIP effectively. Prior to the invasion, most clients had accessed SIP at sites established by harm reduction organizations. With the installation of roadblocks, however, many of these sites became inaccessible to PWID who were afraid to interact with soldiers. Many clients in legal possession of methadone were arrested at these sites due to miscommunication and insufficient knowledge among soldiers. Furthermore, in Russian-controlled regions where Russia took control of the government, OAMT programs became illegal, causing additional challenges for PWID.

*Women are afraid to walk through roadblocks… A lot of guys are afraid too. Especially if they aren’t feeling well, neuroses begin … they begin to behave suspiciously at the roadblock, and then they [the soldiers] undress them, look at injection marks, and check if there are any drugs. So, such people avoid roadblocks”* (Kherson, Russian-controlled).

*“Then moving around with methadone in your pocket, even if you had a permit, the territorial defense [staff] … didn't all know what methadone was. There were a lot of questions, there were a lot of arrests”* (Poltava, proximal destination).

*“Clients are being seriously searched at roadblocks. Searches are done because they are suspicious persons… If a person [has been injecting drugs] for half of his lifetime, then he naturally looks suspicious”* (Kherson, Russian-controlled).

SSPs in regions with roadblocks attempted to accommodate clients by delivering SIP directly to clients’ homes; however, SSP workers also encountered challenges at roadblocks while making these deliveries, further straining supply. Workers were subjected to inspections of their vehicles, precipitating confusion when soldiers found high quantities of SIP and harm reduction supplies. To avoid confiscation of supplies and/or arrest, SSP workers carried documentation to show they were authorized to make these deliveries. Still, some soldiers were reluctant to allow passage of supplies, delaying delivery. Participants reported, however, that once SSP workers had become acquainted with the soldiers working each roadblock, deliveries became smoother.

*“People are afraid of [roadblocks]. But anyway, you get used to it. And the military itself no longer argues with us; in the beginning, it felt odd for them. ‘How is it that he is passing through with such a large load of syringes?’ Well, ‘I'm a volunteer’—you show the sheet and explain the situation. We confirm everything and even go to the roadblock to sort it all out”* (Kherson, Russian-controlled).

#### Cellular network and internet disruptions

In Russian-controlled areas, disruptions to cellular networks and the Internet further restricted the capacity of SSPs to efficiently supply services to clients. For many harm reduction organizations, maintaining contact with clients via messaging services was a crucial method for coordinating distribution of SIP. In Russian-controlled regions, this method of communication largely vanished when Russia took government control of the region. According to one SSP worker in Kherson, Russian occupiers disabled all Ukrainian networks and required individuals in the region to purchase cellular plans from Russian providers. Most clients lacked the funds to purchase these new cellular plans. For the limited number who could, they received brand-new phone numbers that harm reduction organizations did not have recorded in their databases, preventing contact with these individuals.

#### SIP and funding delivery

In Russian-controlled and frontline regions, participants reported significant disruptions to delivery of funding and SIP, restricting SSPs’ capacity to provide clients with sufficient supplies. Several SSPs cut the quantity of SIP that was distributed, reportedly due to issues with SIP delivery that arose due to conflict in these and neighboring areas.

*“In the region where I work, it turned out that we remained in Russian-controlled territory. We had blown up bridges around our town. And there was no access at all. No humanitarian aid vehicles could pass, I mean, absolutely nothing”* (Kaharlyk, Kyiv Oblast; frontline).

Other participants reported that their organizations had substantially reduced the quantity of SIP distributed during the war due to delays in delivery of SIP to harm reduction organizations. This is confirmed in Figure 1.

*“The only issue now is quantity. We can’t give more than five syringes to a person … Now it’s two [per day], but before it was ten”* (Kaharlyk, Kyiv Oblast; frontline).

*“It is clear that [42 syringes per month] is not enough for [the clients]”* (Chernihiv; Russian-controlled).

### Quantitative analysis: the SyrEx database

**Figure 2:**
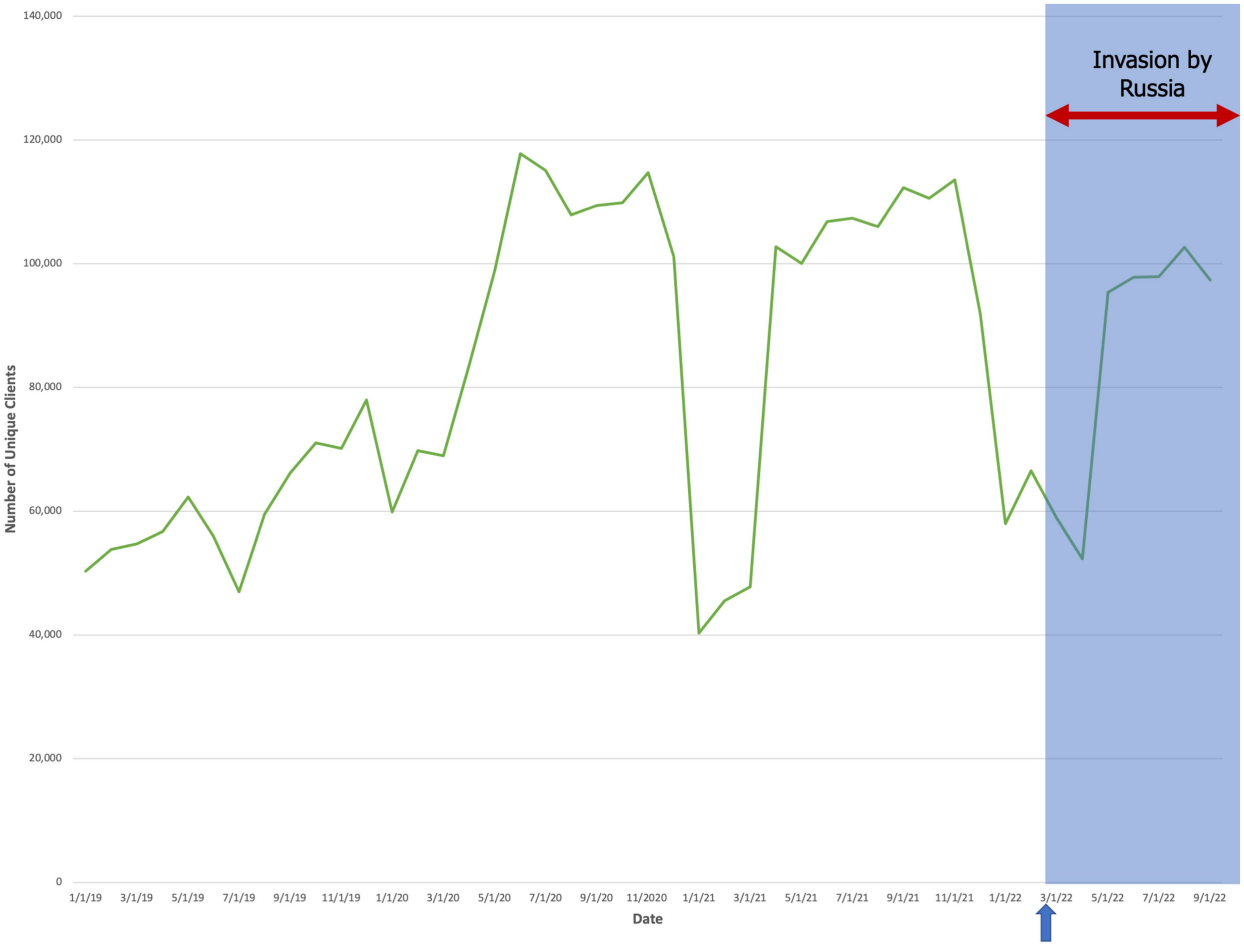
The aggregate quantity of unique clients over time (mm/dd/yyyy) who accessed at least one preventive service at sites across Ukraine. The arrow and shaded blue box denote the start date of the Russian invasion (24 February 2022).

A quantitative analysis of the number of unique clients receiving at least one preventive service per month showed that, overall, the Russian invasion of Ukraine led to a reduction in the number of unique clients per month (Figure 2). Between September 2021 and September 2022, the number of unique clients declined by 13.3% from 112,253 to 97,349. In addition, the data for all Ukrainian harm reduction programs showed that the quantity of unique clients declined during each winter (January through March) from 2019 through 2021. This same trend was observed in 2022, indicating that the initial onset of the Russian invasion did not impact the quantity of unique clients, which was already low due to the winter-time trough. However, the *duration* of this trough in 2022 was longer than for prior years, with relatively low quantities of unique clients being recorded into April and May. This indicates likely evidence of war-related disruptions to these services.

**Figure 3:**
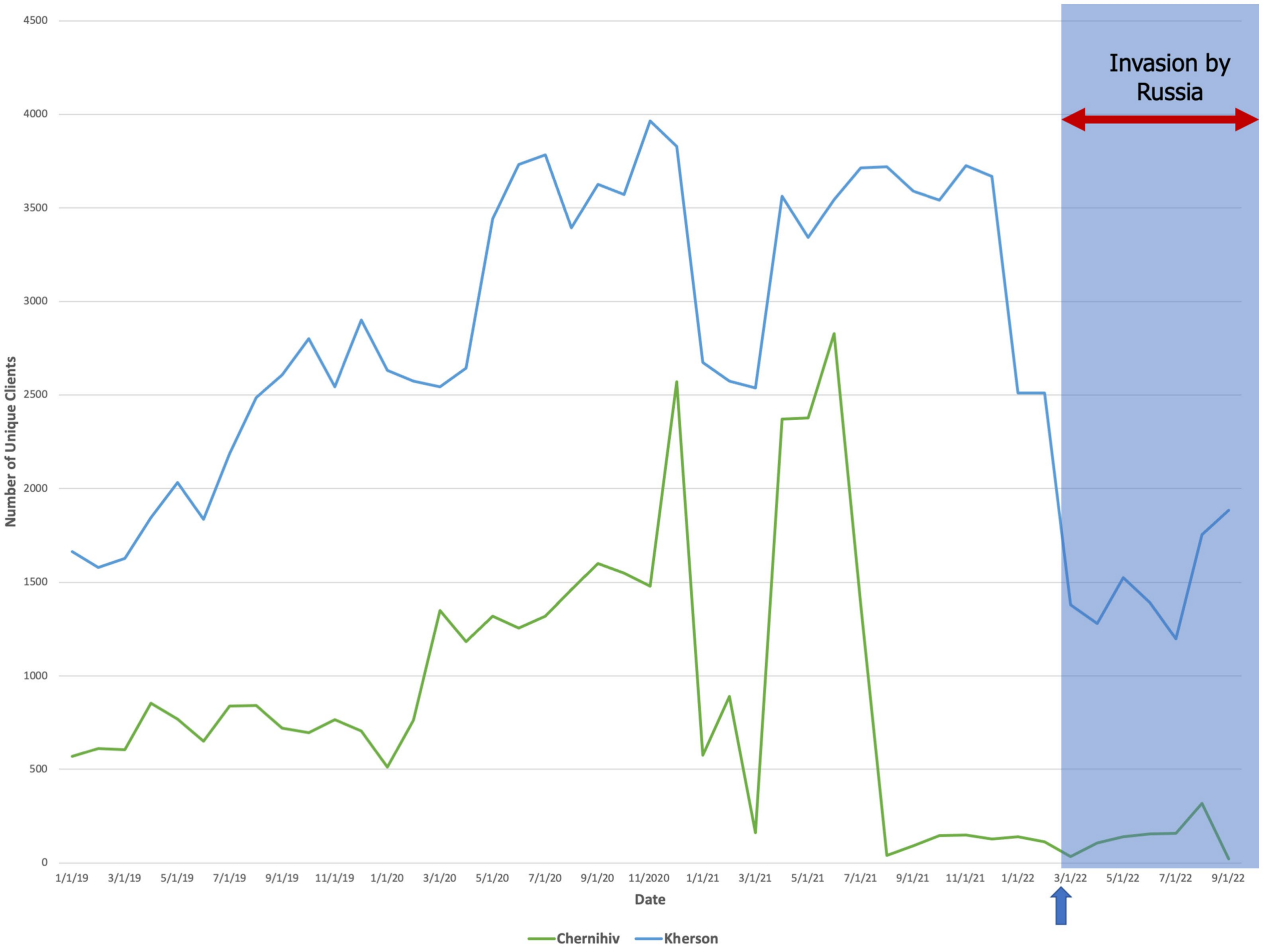
The quantity of unique clients over time (mm/dd/yyyy) who accessed at least one preventive service in temporarily Russian-controlled regions (Chernihiv and Kherson Oblasts). The arrow and shaded blue box denote the start date of the Russian invasion (24 February 2022).

**Figure 4:**
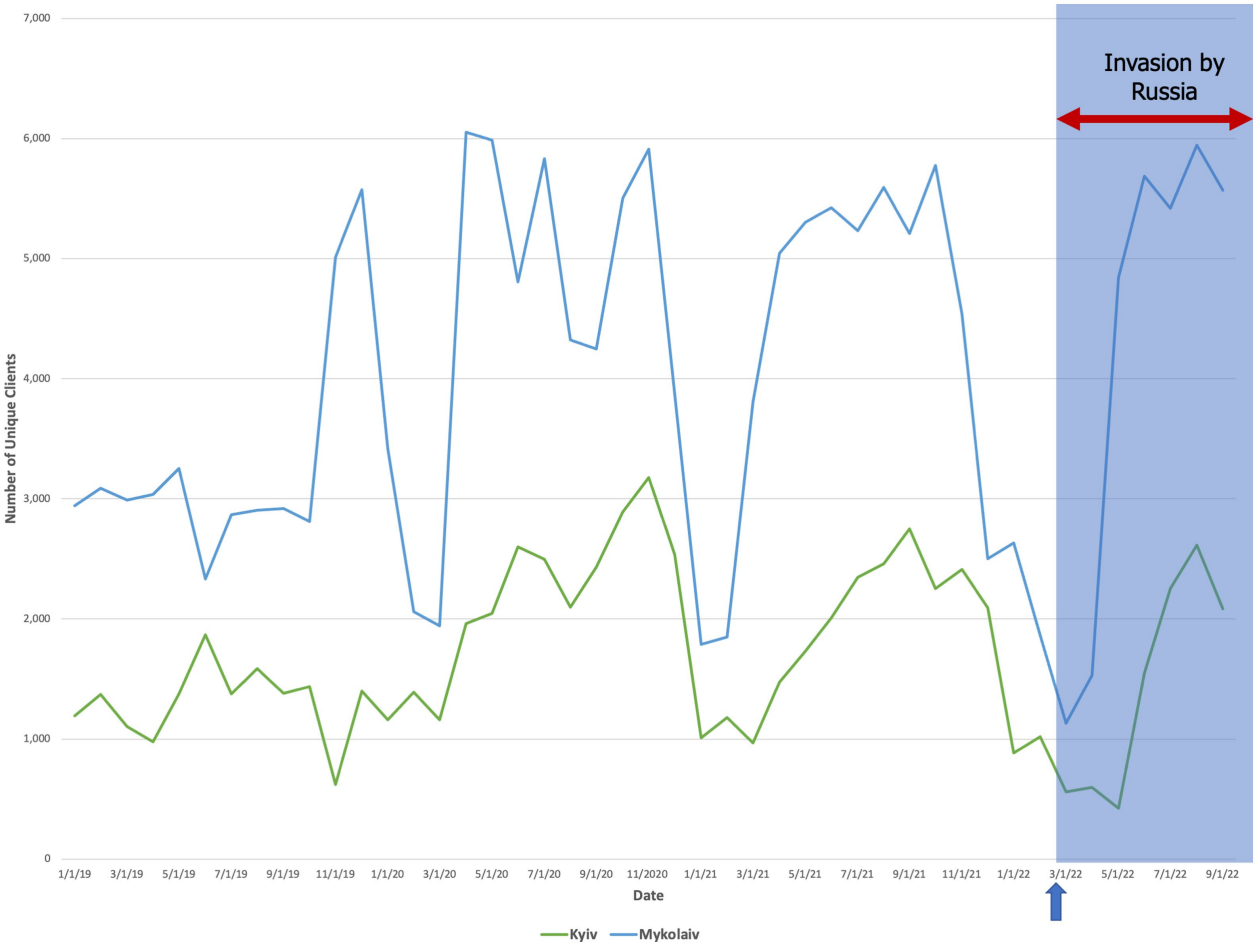
The quantity of unique clients over time (mm/dd/yyyy) who accessed at least one preventive service in frontline regions (Chernihiv and Kherson Oblasts). The arrow and shaded blue box denote the start date of the Russian invasion (24 February 2022).

**Figure 5:**
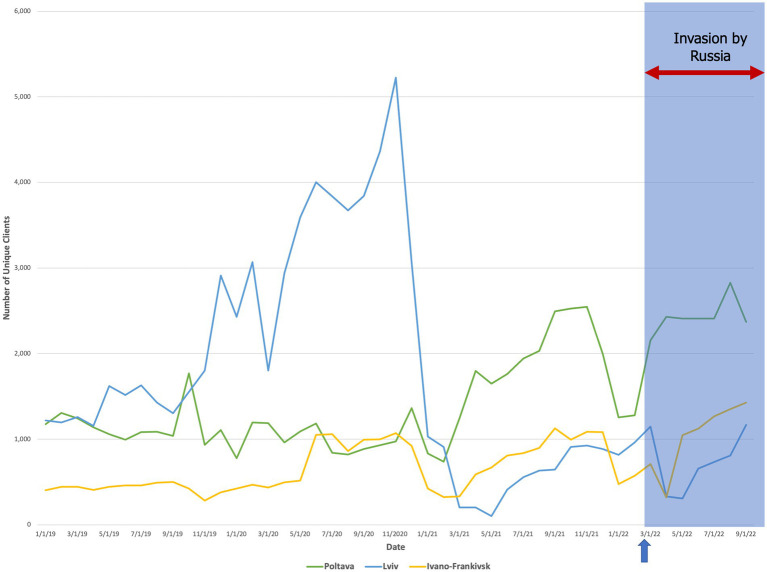
The quantity of unique clients over time (mm/dd/yyyy) who accessed at least one preventive service in destination regions (Polatava Oblast, proximal; Lviv and Ivano-Frankivsk Oblasts, distal). The arrow and shaded blue box denote the start date of the Russian invasion (24 February 2022).

Between oblasts where we completed interviews, we found that for Russian-controlled regions (Kherson Oblast, Chernihiv Oblast), there was a decline in unique clients during the winter that was sustained through September 2022 (Figure 3). In frontline regions (Kyiv Oblast, Mykolaiv Oblast), the quantity distributed to unique clients remained low until May but then rebounded (±5%) to the highest peak from the prior year (Figure 4). Finally, in destination regions (Ivano-Frankivsk Oblast, Lviv Oblast, Poltava Oblast), the results were mixed (Figure 5). While in Ivano-Frankivsk and Poltava Oblasts the peak quantity of unique clients was higher for 2022 than for 2021, this trend was not observed in Lviv Oblast. In Lviv, the reported quantity of clients remained low from January 2021 through September 2022, even though our qualitative interviews suggested an increased workload.

*“More work has been added … people from other regions came to Lviv. That led us to have a lot more work”* (Lviv, distal destination).

This likely indicates that Lviv Oblast’s reporting of clients into the SyrEx database was underestimated.

According to most study participants, the SyRex database was used throughout the war and served as an important source for client linkage, particularly for displaced clients. All clients received de-identified numbers in the database along with a harm reduction card that granted them access to services across all of Ukraine.

*“Those who came from one region to another, with the harm reduction card can receive services at any HIV prevention point. They can find out about prevention centers through the Internet … And they could [also] contact us directly”* (Poltava, proximal destination).

According to other participants, however, reporting to the SyRex database was not consistent, likely explaining the aberrant results observed in Lviv Oblast. For other participants, reporting to the SyRex database stalled amid the conflict due to the limited capacity of harm reduction organizations.

*There was no place to document; we didn’t even have a place to print the documents out [like we did before the war] … We just did it all in a notebook because it was faster* (Obukhiv, Kyiv Oblast, frontline).

When conflict receded in these regions, harm reduction organizations resumed reporting despite these delays.

## Discussion

The Russian invasion of Ukraine poses a major, though not unprecedented, threat to Ukraine’s capacity to continue its response to the HIV epidemic (22). As HIV has become a global pandemic, with many regional HIV epidemics being concentrated in PWID, it is critical to understand the Ukrainian response, which is the first of its type. Before the 2022 invasion, prior events, like the Russian invasion in 2014 and the COVID-19 pandemic, also disrupted HIV services and created learning opportunities (12, 23–27). The COVID-19 pandemic, for example, led to increased psychological distress and heightened drug use among people with HIV (28). During the pandemic, harm reduction services were disrupted in similar ways as those identified during the 2022 Russian invasion (as described in this study), but they were much less pronounced, with most services rebounding after a slight decrease after the initial COVID wave (29). Similarly, the 2014 Russian invasion caused major healthcare disruptions in the occupied areas of Ukraine, likely affecting harm reduction as well (30).

Prior studies have identified similar disruptions to HIV treatment and prevention in Ukraine during the 2022 invasion, though none have focused specifically on harm reduction services to our knowledge. OAMT, a key HIV prevention strategy, has been significantly disrupted, with limited medication supply and rigid guidelines for treating patients that have exacerbated these disruptions (12, 15, 31). As with the healthcare system in Ukraine, access to antiretroviral therapies for treating patients with HIV has also been hindered amid the war (32). Studies on the effects of the war on harm reduction and/or SSPs, however, have been limited. This study therefore offers unique and meaningful insights into other HIV prevention strategies, providing a holistic account of the dynamics of SSPs in Ukraine during the war. Further, it disentangles organizational responses to the war based on context and proximity to actual conflict.

Our findings underline that it is highly important for harm reduction organizations to sufficiently insulate themselves from future disruptor events to ensure continued scale-up of SSPs. Disruption to SSP programs can be directly associated with increased transmission of HIV, making it crucial to continue service delivery (33). To date, Ukrainian harm reduction organizations have been largely successful amid these efforts when accounting for the major disruptions the country encountered with the 2014 Russian invasion of Ukraine and the COVID-19 pandemic (15). During the pandemic, for example, harm reduction organizations transitioned SSPs to mobile delivery and client outreach to telecommunications, enabling them to continue scaling up SSPs despite the risks posed by COVID-19 (29). Still, the challenges faced by harm reduction organizations with managing the higher demand and lower supply of SSP during the 2022 Russian invasion, as described in the results, demonstrates the importance of refining existing methods and implementing new ones to ensure that harm reduction organizations can continue scale-up of SSPs despite the war.

One successful strategy that enabled scale-up of OAMT for HIV prevention during the COVID-19 pandemic (15) and again in the early stages of the war (27) was an effective implementation strategy that applied NIATx (The Network for the Improvement of Addiction Treatment) (34), an evidence-informed collaborative learning strategy with a bundle of implementation tools that can be used to scale-up services. In the same vein, the development of collaborative methods for tracking distribution and other metrics through databases, like the GFATM-funded SyrEx, marks an important innovation to SSP evaluation in Ukraine which can be further leveraged to lower the likelihood of disruptions to scale-up. SyrEx and other databases enable harm reduction organizations to more effectively account for the services that clients require most, which can help guide efficient scale-up of SSPs while accounting for their needs (12). Furthermore, as participants reported in this study, the de-identified client tracking system has helped to improve efficient linkage of clients from one organization to another across the country, which was reported as an innovation for transferring OAMT patients to other regions (12), especially as millions were displaced (13).

SyrEx databases and other methods of data collaboration are therefore crucial for ensuring rapid scale-up during and after disruptor events. First, as proposed by Altice et al. (12) and our study participants, SyrEx can be used in the short term to coordinate more efficient transfers of clients from one harm reduction organization to another amid the high rates of displacement. For example, SSPs can more effectively gauge the needs of new clients by viewing their prior utilization of services in the database. SyrEx can also be leveraged to improve distribution of SIP and funding to harm reduction organizations on an ongoing basis. Particularly during the present crisis, when organizations in Russian-controlled and frontline regions have reported inadequate supplies and funding support while organizations outside these regions had surplus access to supplies and funds, the use of quantitative data to adjust distribution, including time-sensitive transfers to other regions, could be crucial. Furthermore, harm reduction organizations should strive to consistently adhere to data reporting, particularly during periods of crisis, so that allocations and client transfers can be uninterrupted.

Non-governmental organizations (NGOs) that provide all of the harm reduction activities in the country have substantially contributed to cost-effective reductions in transmission of HIV and HCV (35). To sustain these gains, NGOs will need to work in concert with each other across the diverse regions of Ukraine to maintain prevention services to control potentially volatile HIV and HCV outbreaks during the war.

The lessons learned from Ukraine have important applications to other settings, especially where HIV and drug use epidemics are intertwined. First, the shift from opioids to stimulants and other non-opioids based on changes in drug supply, requires that SSPs enhance their services during conflict as OAMT has a limited role. Second, the types of settings that are at risk for such conflicts outside Ukraine are especially tenuous. For example, Russia has already annexed parts of Moldova and Georgia, both with inter-related HIV and opioid epidemics. Therefore, it is crucial for such settings to create preparedness plans for addressing future conflicts (36). Such conflicts extend elsewhere to border disputes between Armenia and Azerbaijan where Russia exerts considerable influence (37). Beyond Eastern Europe and Central Asia, disruptor events like natural disasters, refugee crises, and pandemics could also cause disruptions to SSP service delivery. The COVID-19 pandemic caused major disruptions to SSPs and other harm reduction programs in the U.S. and in most other countries (38). For all countries with SSPs, therefore, it is important that harm reduction staff observe the lessons learned from Ukraine. Some likely steps would be to stock markedly higher levels of sterile injection equipment and to facilitate a smoother transition to large distribution of SIP while concurrently promoting secondary distribution practices where some PWID distribute to their social and injection networks. As suggested in the case of Ukraine, facilitation of increased collaboration and streamlining of client and related data could also have a significant impact on reducing the likelihood of SSP interruption (12).

### Limitations

Though this study attempts to summarize the response of harm reduction organizations to barriers during the Russian invasion, it may not provide the full picture of the situation in Ukraine. Rather, it is based on the limited, available quantitative data and in-depth interviews that were performed with purposively selected participants across all regions of interest. Of note, this study does not include data from the Donetsk, Luhansk, or Crimea regions. This was due largely to logistical constraints, as it is very difficult to establish contact with SSPs and/or clients in these areas, which have been under Russian occupation since 2014. Moreover, this study was more focused on the specific response to the 2022 Russian invasion rather than the 2014 invasion.

## Conclusion

Maintenance and scale-up of SSPs in Ukraine is critical to preventing the spread of HIV and HCV for PWID. These programs not only distribute sterile SIP but also support and navigate clients to other services like OAMT that are crucial for overdose prevention and primary and secondary HIV prevention. Findings here demonstrate that the war has led to pressing and disparate needs across three different zones within Ukraine—Russian-controlled, frontline, and destination. A targeted response should be tailored to each of these prototypical regions with an effective implementation strategy to ensure service continuity. Throughout the country, a national response that adheres to the supply and demand needs of SSPs for each harm reduction organization is critical to sustaining scale-up of SSPs despite the Russian invasion. Mobilization of existing resources like the SyrEx database can be particularly helpful for ensuring an improved response and recovery in the SSP space. These lessons from Ukraine should be applied across other countries that are similarly encountering concurrent HIV and drug injection epidemics.

## Data availability statement

The datasets presented in this study can be found in an online repository. The repository can be accessed at the following link: https://doi.org/10.17605/OSF.IO/BF4SK.

## Ethics statement

The studies involving humans were approved by Yale University and the Ukrainian Institute of Public Health Policy. The studies were conducted in accordance with the local legislation and institutional requirements. The ethics committee/institutional review board waived the requirement of written informed consent for participation from the participants or the participants’ legal guardians/next of kin because due to the low-risk nature of the study and the ongoing conflict in Ukraine, both IRBs endorsed collection of verbal consent from participants.

## Author contributions

BN: writing-original draft, conceptualization, methodology, investigation, visualization, and formal analysis DB: conceptualization, methodology, investigation, visualization, writing-review and editing, and formal analysis IP: conceptualization, validation, and resources RI: validation and methodology ZI: validation, resources, and conceptualization. FA: supervision, conceptualization, and validation. All authors contributed to the article and approved the submitted version.
