# Supplementary material for: Early disruptions to syringe services programs during the Russian invasion of Ukraine
Source: Front Public Health. 2023 Nov 21;11:1229057. doi: 10.3389/fpubh.2023.1229057 (PMC10702598; doi:10.3389/fpubh.2023.1229057)
Supplement: Supplementary file 1 [file Data_Sheet_1.docx]

Supplementary Material

Early disruptions to syringe services programs during the Russian invasion of Ukraine

Benjamin M. Nikitin, Daniel J. Bromberg, Iryna Pykalo, Roman Ivasiy, Zahedul Islam, Frederick L. Altice

*** Correspondence:** Benjamin M. Nikitin: [ben.nikitin@yale.edu](mailto:ben.nikitin@yale.edu); Iryna Pykalo: [pykalo@eiphp.org](mailto:pykalo@eiphp.org)

**Interview Guide (original, Russian):**

**Преамбула:**

Мы понимаем, насколько вам сейчас нелегко. Мы знаем, что вы очень заняты, и безмерно благодарны за то, что вы нашли время, чтобы рассказать нам о своей работе.

**Что произошло?**

Нам очень интересно узнать, как изменились ваши программы в связи с текущим кризисом. Расскажите пожалуйста о работе, которую вы делаете. Например:

а. Чем сегодняшняя работа отличается от той работы, которую вы выполняли до начала конфликта? (Слушайте внимательно — раздаются ли шприцы в том же количестве, что и раньше? Если есть изменения, чем они вызваны? И  как выглядит поставка наркотиков сегодня — в отчаянии ли  клиенты? Как они справляются? Есть ли доступ к ЗПТ (Заместительная поддерживающая терапия)?

б. Что конкретно вам нужно сегодня изменить в вашей работе? (Слушайте внимательно– помощь в подключении ВПЛ к ЗПТ, помощь с документами и паспортами ВПЛ, жильем)

**Как это произошло?**

Как это произошло? (Подскажите: Как это стало возможным? Расскажите о новом законодательстве, если такое существует. Что это за законодательство? Что послужило толчком? Кто инициировал дискуссию? Изменились ли разговоры со временем? Произошло ли это сразу после начала конфликта? Кто участвовал в этом переходе?)

**Как дела?**

Как выглядит ваш обычный рабочий день, чем занимается ваш персонал? (Подсказка: в каком вы сейчас находитесь состоянии?) Как вы сообщаете пациентам, что им необходимо получить, иглы/шприцы?

 Каков ваш общий план распределения игл и шприцев?

Что идет хорошо? (Подскажите, если нужно: что можно улучшить?)

Как у вас обстоят дела с иглами/шприцами? (Подсказка: Как вы планируете решить проблему нехватки шприцов, если таковая возникнет?

Какие организации оказывают вам поддержку? (Подсказка: ЦГЗ (Центр громадського здоровя), Глобальный фонд, коллеги и т. д.)

Какие успехи вы могли бы отметить?

Что бы вы хотели улучшить в своей работе?

Какая поддержка вам нужна, чтобы лучше выполнять свою работу?

 Чему мы должны научиться из полученного опыта?

 Чему вы научились, благодаря текущей ситуации?? (Подсказка: изменили ли бы что-либо в вашей работе, если бы у вас была такая возможность?

**Interview Guide (translated, English):**

**Preamble:**

We can’t imagine how challenging things must be right now. We know how busy you must be and thank you for taking the time to bring us up to date with all the work you are doing. First, how are you doing personally?

**What happened?**

We are very interested in hearing how your programs have evolved in response to the crisis. Would you mind giving a general overview of your current work? Some probes:

a. How does this work differ relative to before the conflict? (listen for – are they distributing syringes to the same extent as before – more? or less? and why – what does the drug supply look like – are their clients feeling desperate? How are their clients coping? Do more of their clients want to access to OAT?

b. What sorts of things have you not been able to do and what kinds of new things are you having to do that you were not doing previously (listen for – helping connect IDPs to OAT, assist with IDP documents and passports, housing)

**How did this come about?**

How did this happen?  (Prompt: what made this possible? What legislation? What was the trigger? Who initiated the discussion? Have the conversations changed over time?

Did this happen all at once? Who was involved in this transition?)

**How is it going?**

What does your average day look like for the program – the staff in the program?

(Prompt: What is it really like to be you right now?)

How are you communicating with patients to let them know about picking up needles/syringes?

What is your general distribution plan for needles and syringes?

What is going well? (Prompt, if necessary: What could be going better?)

How is your supply of needles/syringes? (Prompt: Are you concerned about running out? How are you planning to address that?)

What kind of support are you receiving? (Prompt: From CPH, the Global Fund, colleagues, etc)

What kinds of things that you are doing are going really well?

What kinds of things do you wish you had time to do or would like to do better?

What kinds of supports do you need in order to do your job better?

What is frustrating you in being able to do your job the way you really want to?

What should we learn from what is happening?

What have you learned in responding to this emergency? (Prompt: Would you do things differently than how they have evolved?)
